# Microbiomes of American Oysters (*Crassostrea virginica*) Harvested from Two Sites in the Chesapeake Bay

**DOI:** 10.1128/genomeA.00729-17

**Published:** 2017-07-27

**Authors:** Sylvia Ossai, Padmini Ramachandran, Andrea Ottesen, Elizabeth Reed, Angelo DePaola, Salina Parveen

**Affiliations:** a Department of Agriculture, Food and Resource Sciences, University of Maryland–Eastern Shore, Princess Anne, Maryland, USA; b Office of Regulatory Science, Center for Food Safety and Applied Nutrition, Food and Drug Administration, College Park, Maryland, USA; c Angelo DePaola Consulting, Coden, Alabama, USA

## Abstract

In this study, we used 16S rRNA gene amplicons to describe the bacterial microbiota associated with oysters (*Crassostrea virginica*) and seawater collected from two sites in the Chesapeake Bay. The dominant bacterial groups included those belonging to the order *Pelagibacteraceae*, family *Enterobacteriaceae*, and genus *Synechococcus*. The microbiomes varied among oysters from the same site and between the two sites and months.

## GENOME ANNOUNCEMENT

American oysters (*Crassostrea virginica*) are one of the most important commercial catches in the Chesapeake Bay (1) and, as is well documented, act as important ecosystem engineers in bays and estuaries (2). A single adult oyster can filter up to 50 gal of water per day, playing a significant role in water quality. As filter feeders, oysters accumulate particles from surrounding waters, including a wide range of microorganisms that can include human pathogens, such as bacteria (e.g., *Vibrio* spp.) and viruses (e.g., *Norovirus*) (2–4). Infections from these pathogens can cause serious health issues (5, 6), which makes an improved understanding of the oyster microbiome an important public health and food safety objective (2, 3).

Much work has effectively described the prevalence of human pathogens, such as *Vibrio* spp. (7–10), associated with oysters. Additional culture-independent profiling of bacterial species associated with oyster microbiomes will continue to improve our understanding of risk factors and mitigation strategies.

We used a metagenomic approach (16S rRNA gene amplicons) to characterize the bacterial composition of *C. virginica* and water from the Chesapeake Bay. A total of 20 oysters and 12 water samples were collected between May and June 2016 from two sites (Manokin River and Chester River) in the Chesapeake Bay. At each collection event, five oysters and 1 L of water were collected from each site. All collected samples were transported to the University of Maryland–Eastern Shore and processed within 4 h of collection (9).

Oysters were blended using a sterile Waring blender (Waring, Stamford, CT, USA) to produce homogenized samples (9). Water subsamples (3) containing 200 mL each were filtered using a 0.22-µm Sterivex filter (Millipore, Billerica, MA, USA) (8). All processed samples were stored at −80°C until DNA extraction (within 48 h). Genomic DNA from oyster homogenates and water samples were extracted using the DNeasy blood and tissue kit (Qiagen, Germantown, MD, USA) and the Powersoil DNA isolation kit (Mo Bio Laboratories, Inc., Carlsbad, CA, USA), respectively. The 16S rRNA gene amplification targeted the V1-to-V3 region.

The bacterial diversity observed in the oyster microbiome suggests that it hosts a diverse and dynamic bacterial community that is both site and temporally dependent. Temporal variation was evident with the family *Pelagibacteraceae*. Relative abundances of *Pelagibacteraceae* were 13.3% and 28.5% in oysters in May in Chester and Manokin, respectively. In June, the relative abundances were only 0.2% and 0.1% in oysters from Chester and Manokin, respectively. *Synechococcus* had relative abundances of 35.7% and 16.2% in oysters from the Chester and Manokin sites, respectively, in the month of June. Also in June, *Enterobacteriaceae* had relative abundances of 57.5% and 25.5% in oysters from the Manokin and Chester sites, respectively. *Synechococcus* (25.1%) and *Pelagibacteraceae* (23.5%) predominated in the water in Manokin and Chester, respectively, in June. Surprisingly, the average relative abundance of *Vibrio* spp. was never higher than 2.5% in either site or month. Additional biological replicates and a wider range of biogeographic and temporal sampling will continue to improve our understanding of bacterial microbiota associated with oysters in the Chesapeake Bay.

### Accession number(s).

All data have been deposited in NCBI GenBank under accession numbers SRR5429782 through SRR5429811 (BioProject ID: PRJNA381771).
